# *CORR* Insights^®^: External Rotation Immobilization for Primary Shoulder Dislocation: A Randomized Controlled Trial

**DOI:** 10.1007/s11999-014-3466-4

**Published:** 2014-02-01

**Authors:** Brett D. Owens

**Affiliations:** Department of Orthopaedics, Keller Army Community Hospital, 900 Washington Rd., West Point, NY 10996 USA

## Where Are We Now?

Early treatment of first-time anterior glenohumeral dislocations still elicits controversy and active investigation. In 2001, Itoi and colleagues [3] introduced the concept of bracing in external rotation with increased tension of the subscapularis resulting in a coaptation of the avulsed capsulolabral complex back to its bony bed, which allowed for healing in a reduced position. After showing proof of concept with an MRI study [3], this group moved forward with randomized trials comparing external rotation bracing for 3 weeks, with 3 weeks in a standard internal rotation sling [1, 2]. Their initial trial enrolled 40 patients with a mean age of 39 years (range 17 to 84 years) and found a significant reduction in recurrent dislocation in the external rotation group [1]. A subsequent trial included randomized 198 patients representing a slightly younger cohort (mean ages 35 and 37 years), and had similar findings [2]. The authors included a subgroup analysis, and found that their results also held true for patients younger than 30 years of age.

Despite promising studies by this group in an Asian population, other investigators have not been able to reproduce these results [4, 5]. In an Israeli study [4] of 51 subjects with a mean age of 20 years (range 17 to 27 years), 78% of whom were soldiers, no decrease in recurrent dislocation was noted with external rotation bracing. Similarly disappointing results for recurrent dislocation were seen in a well-executed randomized controlled trial of 188 patients in Norway [5]. This study by Liavaag and colleagues [5] also enrolled a younger cohort, with a mean age of 26 years (range 16 to 40 years); they too failed to find differences in Western Ontario Shoulder Instability (WOSI) scores between groups as well as recurrent subluxation. They also included an age subgroup analysis, again, not showing any differences between groups. A recent meta-analysis [6] utilized pooled data from five randomized controlled trials and close to 500 patients, but did not demonstrate evidence of efficacy of external rotation bracing for reducing recurrent dislocation.

## Where Do We Need To Go?

The study by Whelan and colleagues [7] is the first randomized controlled trial to evaluate the efficacy of external rotation for the acute management of first-time anterior glenohumeral dislocation in a North American population. Whelan and colleagues enrolled a young cohort with a mean age of 23 years (range 14 to 35 years), and extended sling or brace treatment to 4 weeks instead of 3 weeks. While the study may be underpowered with 60 total patients, the results are consistent with the Finestone [4] and Liavaag [5] reports. Whelan and colleagues reported that six of 27 subjects in the external rotation group and eight of 25 in the internal rotation group experienced a recurrent dislocation (not significant). However, 10 subjects in both groups experienced significant instability when including multiple recurrent subluxation events. There was no improvement in WOSI scores, but there was an improvement in American Shoulder and Elbow Surgeons scores favoring the external rotation group that reached statistical, but not clinical significance. This work adds to the growing body of literature challenging the effectiveness of external rotation bracing for preventing recurrent instability in first-time anterior dislocation.

Going forward, our field needs to definitively determine whether external rotation bracing is effective, as there appears to be two rather divergent sets of data — those from Itoi’s group, which included some very well done studies, and the results of the many studies that have been performed since then, which did not replicate Itoi’s intriguing findings.

## How Do We Get There?

It is unclear what accounts for the discrepancies in the results reported by Itoi and others. While the Itoi cohorts were somewhat older than many of the others, one of Itoi’s studies provided a subgroup age analysis and found the effect of external rotation bracing to hold true in the subgroup younger than 30 years [2]. There may also be population differences related to cultural norms or athletic activities that may partially explain these discrepancies. Most of the studies performed to date have experienced difficulty with patient compliance and have incorporated mixed populations of athletes and nonathletes.

While there may be efficacy for a subgroup of patients experiencing first-time anterior glenohumeral dislocation, this has yet to be consistently shown, and identifying precisely which patients may benefit — if any — remains a challenge. In the subgroup at greatest risk for recurrence (young, contact athlete), there has been a consistency of data supporting surgical stabilization compared with nonoperative management. A more relevant debate than external rotation bracing versus internal rotation sling may be which type of surgical intervention results in the most reliable result, a high likelihood of return to function, and a low risk of recurrent instability. It is critical that our research consider the full array of endpoints that our patients consider important, including ensuring that all recurrent instability events are reported (including subluxations), in addition to using sensitive subjective outcome scores such as the WOSI, and reporting on activity level measures instead of solely relying on the outcome of recurrent dislocation.
